# Amaryllidaceae plants: a potential natural resource for the treatment of Chagas disease

**DOI:** 10.1186/s13071-021-04837-9

**Published:** 2021-06-26

**Authors:** Nieves Martínez-Peinado, Nuria Cortes-Serra, Luciana R. Tallini, Maria-Jesus Pinazo, Joaquim Gascon, Jaume Bastida, Julio Alonso-Padilla

**Affiliations:** 1 Barcelona Institute for Global Health (ISGlobal), Hospital Clinic-University of Barcelona, 08036 Barcelona, Spain; 2 Departament de Biologia, Sanitat i Medi Ambient, Facultat de Farmàcia I Ciències de L´Alimentació, Universitat de Barcelona, 08028 Barcelona, Spain; 3 Graduate Program in Pharmaceutical Sciences, Federal University of Rio Grande Do Sul, Porto Alegre - RS, 90610-000 Brazil

**Keywords:** Chagas disease, *Trypanosoma cruzi*, Amaryllidaceae, Extracts, Phenotypic assays, Cytotoxicity

## Abstract

**Background:**

Chagas disease is a neglected zoonosis caused by the parasite *Trypanosoma cruzi*. It affects over six million people, mostly in Latin America. Drugs available to treat *T. cruzi* infection have associated toxicity and questionable efficacy at the chronic stage. Hence, the discovery of more effective and safer drugs is an unmet medical need. For this, natural products represent a pool of unique chemical diversity that can serve as excellent templates for the synthesis of active molecules.

**Methods:**

A collection of 79 extracts of Amaryllidaceae plants were screened against *T. cruzi*. Active extracts against the parasite were progressed through two cell toxicity assays based on Vero and HepG2 cells to determine their selectivity profile and discard those toxic to host cells. Anti-*T. cruzi*-specific extracts were further qualified by an anti-amastigote stage assay.

**Results:**

Two extracts, respectively from *Crinum erubescens* and *Rhodophiala andicola*, were identified as highly active and specific against *T. cruzi* and its mammalian replicative form.

**Conclusions:**

The results retrieved in this study encourage further exploration of the chemical content of these extracts in search of new anti-*T. cruzi* drug development starting points.

**Graphic abstract:**

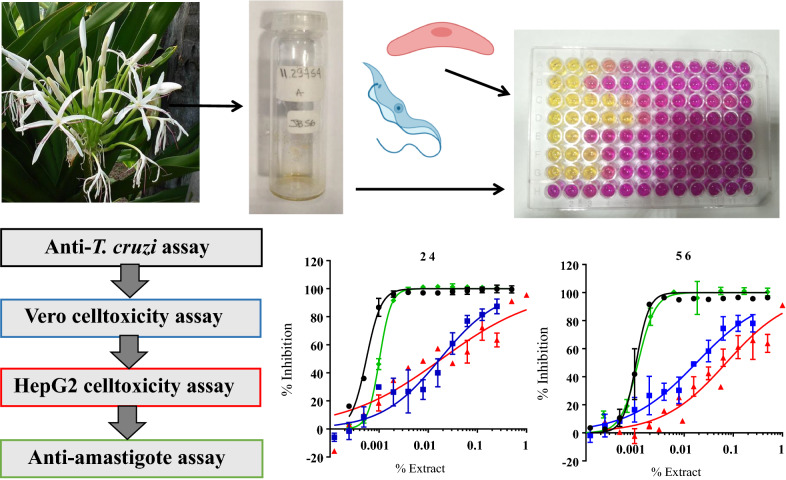

**Supplementary Information:**

The online version contains supplementary material available at 10.1186/s13071-021-04837-9.

## Background

The parasite *Trypanosoma cruzi* is the etiological agent of Chagas disease, a debilitating zoonosis affecting over six million people worldwide [1]. It was discovered over 110 years ago, but remains an important public health problem in Latin America, where it exerts its highest burden [1]. In the past few decades, the disease impact has been globalized to non-endemic regions due to population movement [2].

In the disease course, there is first an acute phase that goes undiagnosed and untreated because it is mostly asymptomatic. Without treatment, however, it evolves to an indeterminate chronic phase throughout which about 30% of those chronically infected will develop damage to cardiac and/or digestive tissues, which can lead to death [3].

There is no vaccine available, but there are two chemotherapeutic treatments for *T. cruzi* infection: benznidazole (BNZ) and nifurtimox (NFX). Both have good efficacy when administered early after infection and are well tolerated by infants. However, at the chronic stage, which is when a diagnosis is usually obtained, their efficacy diminishes [3], and the appearance of toxic side effects is common [4]. In addition, treatment response may vary according to the parasite strain, and drug resistance has been observed [5]. Thus, safer and more efficacious drugs for the treatment of Chagas disease are needed.

Upon clinical failure of azoles (posaconazole and E1224) [6], the pipeline of new chemical entities under clinical trial is limited to fexinidazole [7] and amiodarone [8]. Most presently ongoing trials are based on the evaluation of alternative regimens and doses of already known drugs [4, 9], so new chemical entities would be very welcome in the field. For this purpose, natural products represent a rich source of biologically active substances [10–12]. They offer a wealth of bio-structural information that can be used to guide drug discovery and molecular design [10–12]. In fact, around 60% of the drugs available nowadays derive from natural products [13].

In an attempt to find potential therapies for Chagas disease, the search for new compounds extracted from plants has intensified in the past decade [14]. Almost 400 species belonging to more than 100 plant families have been evaluated against *T. cruzi* [14]. Most of the reports have studied the activity of isolated alkaloids, flavonoids, cathechins, lignans, diterpenes and sesquiterpenes [14]. The extraction process for these chemical entities can involve the use of one or more solvents, as well as a single and/or various parts of the plant. Usually, the parts of the plants evaluated are the ones used in traditional treatments or those described to carry higher concentrations of the known active compounds [14].

Recently, members of the family Amaryllidaceae have attracted considerable attention due to their alkaloid composition, with unique structural features and multiple types of biological activity [15, 16]. Isoquinoline-type alkaloids found in Amaryllidaceae plants are classified into eight groups: galanthamine, lycorine, crinine, haemanthamine, homolycorine, narciclasine, tazettine and montanine [16]. Each plant species produces a different mixture of alkaloids due to differential expression of the required biosynthetic enzymes, which have their own substrate specificity [17]. Moreover, variation in the alkaloid composition has been observed within the same plant species collected from different locations [18], and between different biological tissues of the same plant [15]. Amaryllidaceae plants have been used as part of traditional treatments of fever, swelling, cancer or malaria for centuries [19]. They have subsequently been studied for their potential application as a source of anticancer, anti-inflammatory, antimicrobial and anticholinesterase activity [15]. In fact, Amaryllidaceae alkaloid galanthamine, an acetylcholinesterase inhibitor, received approval by the Food and Drug Administration to treat Alzheimer’s disease [20]. Thinking of their anti-parasitic use, some Amaryllidaceae alkaloids have shown promising results against *Plasmodium falciparum*, *Leishmania donovani*, *Trypanosoma brucei* and *T. cruzi* [21–26]. In a recent study, we reported that alkaloid hippeastrine showed specific activity against the latter and its amastigote form [23]. Now, with the aim to identify novel drug development starting points from natural products, we have screened a collection of 79 extracts from Amaryllidaceae plants for their anti-*T. cruzi* activity.

## Methods

### Amaryllidaceae plant extracts

Plants were collected and supplied under the framework of the CYTED BIFRENES project network (ref. 416RT0511). Alkaloids are the principal active constituent of Amaryllidaceae, and the extraction procedure explained below was devised to solely keep their alkaloid fraction. Unless otherwise indicated, alkaloid extracts were obtained from aerial parts or bulbs through acid–base extraction. Each species was collected and dried for 7 days at 40 ºC. Samples were ground and the powders macerated with methanol (3 × 100 mL) at room temperature for 3 days. The mash was filtered and the organic solvent was taken to dryness under reduced pressure to obtain the crude extract. These extracts were acidified to pH 3 with sulfuric acid (2%, volume/volume [v/v]) in order to remove the neutral material with diethyl ether. Aqueous solutions were then basified up to pH 10 with ammonium hydroxide (25%, v/v) to obtain the alkaloid extract with ethyl acetate. All alkaloid extracts were dried using reduced pressure. Dried material of each extract was solubilized with 100 µL of dimethyl sulfoxide (DMSO) and used in the experiments. In all cases, the final DMSO percentage per well was below 0.5%. The collection of alkaloid extracts is shown in Additional file 1: Table S1 and Additional file 2: Table S2.

### Host cell and *T. cruzi* parasite cultures

Vero (green monkey kidney), LLC-MK2 (rhesus monkey kidney) and HepG2 (human liver) epithelial cells were cultured as described previously [23]. Mammalian infective stages of *T. cruzi* Tulahuen β-gal parasites (discrete typing unit VI) were maintained in LLC-MK2 cells and purified as previously described [27].

### Anti-*T. cruzi* primary assay

The assay protocol followed has been described previously [23]. Briefly, Vero cells were diluted to 1 × 10^6^ cells/mL. In parallel, isolated trypomastigotes were diluted at the same concentration, and both cell types mixed at a 1:1 ratio so that 50,000 Vero cells and 50,000 trypomastigotes were placed in each well (multiplicity of infection or MOI = 1) [23]. Before that, extracts had been poured into the assay plates at a starting volume of 2 or 1 µL, followed by further threefold dilution fashion. BNZ was used as positive control of drug growth inhibition, and each plate contained its own negative (Vero cells and parasites) and positive controls (trypomastigote forms alone) [23]. Plates were incubated for 4 days at 37 ºC and the readout was made with chlorophenol red-β-D-galactoside (CPRG) substrate, as described previously [23, 28].

### Anti-*T. cruzi* amastigote assay

As previously described [29], Vero cells were infected in bulk. They were seeded in T-175 flasks (5 × 10^6^ cells/flask) and cultured for 24 h. Then, cells were washed with PBS, and free-swimming trypomastigotes (1 × 10^7^ trypomastigotes per flask; MOI = 1) were added for 18 h. Infected cell monolayers were washed in PBS and detached from the flask. Cells were counted and diluted to a concentration of 5 × 10^5^ cells/mL, before adding 100 µl per well to test plates already containing the extracts. In all cases we included BNZ as control drug, and each plate contained its own negative (Vero cells and parasites) and positive controls (Vero cells) [29].

### Cell toxicity assays

Vero and HepG2 cell toxicity assays were performed as described previously [23]. Briefly, cultures were washed with PBS, detached, and viability checked with Trypan blue before adding 100 µl per well at the corresponding concentration [23]. All assay plates contained their own negative and positive controls [23, 31]. Upon addition of biological reagents, they were incubated at 37 ºC for 4 days (Vero) or 2 days (HepG2). AlamarBlue was used for assay readout, as described previously [23].

### Data analysis

Absorbance and fluorescence values were normalized to the controls [30]. IC_50_ and TC_50_ values were determined with GraphPad Prism 7 software (version 7.00, 2016) using a non-linear regression analysis model [23]. Extracts IC_50_ and TC_50_ mean and standard deviation (SD) values are presented as parts per million (ppm) [32]. IC_50_ and TC_50_ values as ppm of the reference drugs BNZ and digitoxin (DTX) were calculated considering their average micromolar values and their respective molecular weights. Occasionally, BNZ IC_50_ and TC_50_ values are provided as average micromolar values with their corresponding SD. In Additional file 1: Tables S1 and Additional file 2: Table S2 we also provide the *R*^2^ fit-adjustment of the extracts’ dose–response curves.

## Results

### Anti-*T. cruzi* activity of extracts from Amaryllidaceae plants

The collection of Amaryllidaceae extracts was screened using a phenotypic assay based on the parasite *T. cruzi* Tulahuen-β-gal and Vero cells as hosts [23]. In every run we included the reference drug BNZ as positive control of drug inhibition. Its mean IC_50_ was 1.55 (standard deviation, SD = 0.05) µM [i.e. 0.40 (0.01) ppm] (Additional file 3: Figure S1A), which correlates with previously reported values [23, 27].

All extracts were tested at least in triplicate following a 1:3 dilution dose–response pattern to calculate their IC_50_ values. Extracts 51, 93, 97 and 117 showed the highest activity against *T. cruzi* with IC_50_ values between 1 and 2 ppm (Additional file 1: Table S1). Since extracts are complex biological mixtures, we considered active those with IC_50_ values within a range 30 × that of BNZ. In agreement with this threshold, we identified 37 active (Additional file 1: Table S1) and 42 inactive extracts (Additional file 2: Table S2).

### Identification of extracts with specific activity against the parasite

In order to ascertain whether retrieved anti-*T. cruzi* activity was indeed specific against the parasite, and to discard any extracts that were toxic to the host cells instead, we performed a secondary cell toxicity assay on Vero cells. BNZ was included in every run as reference, and its average TC_50_ was 267.5 (14.38) µM [i.e. 69.6 (3.74) ppm] (Additional file 3: Figure S1A), correlating with previously reported values [23, 30].

**Fig. 1:**
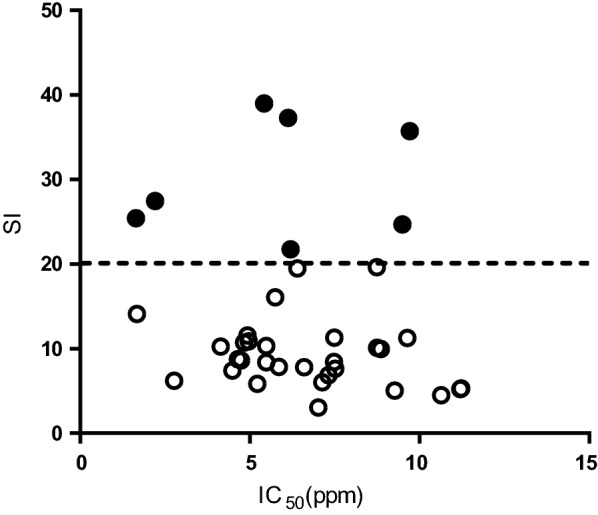
Distribution of active extracts according to their IC_50_ and SI values. Parasite-selective extracts are represented by filled circles and non-selective extracts by empty circles. Horizontal dashed line indicates the threshold of SI > 20

**Fig. 2:**
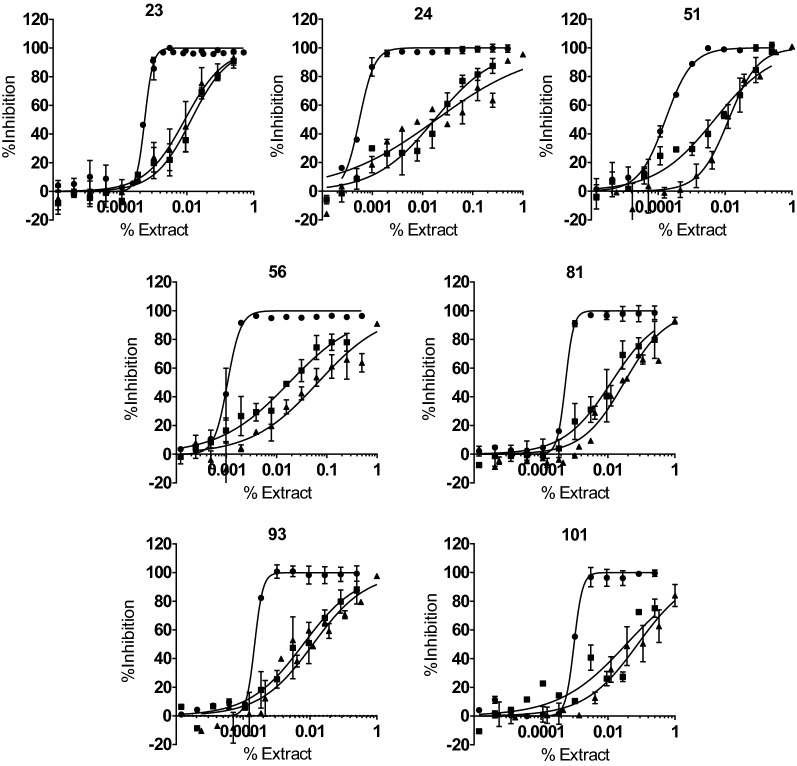
Dose–response curves of selected extracts based on the specificity of their anti-parasitic activity. Anti-*T. cruzi* activity data are represented by circles, Vero cell toxicity data by squares, and HepG2 cell toxicity data by triangles. Graphs represent mean results and SD of at least three independent biological replicates [23, 24]

Twelve out of 37 extracts were less toxic to Vero cells than BNZ (Additional file 1: Table S1). Extracts 24, 56, 81 and 101 had the highest TC_50_ to Vero cells, with values ranging from 211.5 to 346.7 ppm (Additional file 1: Table S1). We established a selectivity index cut-off of 20 × TC_50_/IC_50_ (SI > 20), twice the ratio considered elsewhere, to progress compounds with specific anti-parasitic activity [31]. As a result, the activity of seven of the active extracts was determined as specific against the parasite (Fig. 1). Their dose–response activity curves against *T. cruzi* and Vero cells are shown in Fig. 2.

**Fig. 3:**
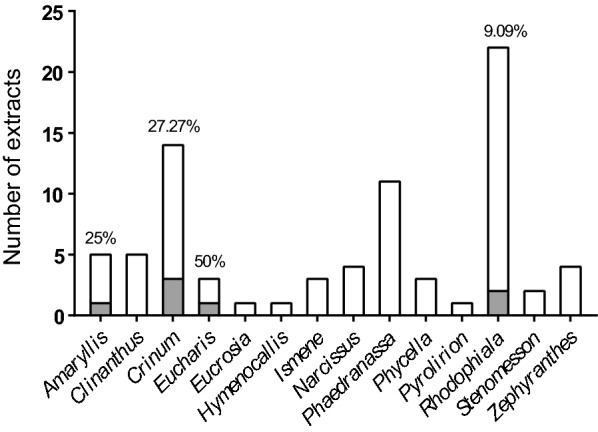
Distribution of anti-*T. cruzi* selective extracts per plant genus. Percentage was calculated as the number of selective extracts (represented in grey) over the total number of extracts within each genus

Remarkably, three of the extracts showed a TC_50_/IC_50_ ratio higher than 30, wherein extracts 24 and 81 presented the highest selective anti-*T. cruzi* activity, with SI values > 37 (Table 1). Among the extracts that showed specific activity against the parasite, those from genera *Rhodophiala* and *Crinum* predominated over the rest, with two extracts per genus (Fig. 3).

**Table 1:** Table displaying IC_50_, TC_50_ and SI values of extracts with specific activity against Vero and HepG2 cell

No. extract	Plant species of origin	Country of collection	Part of the plant*	IC_50_ (ppm)	TC_50_^a^ (ppm)	SI^a^	TC_50_^b^ (ppm)	SI^b^	IC_50_^c^ (ppm)	SI^a,c^	SI^b,c^
BNZ	–	–	–	0.40	69.60	174.0	51.47	128.7	0.53	131.4	97.6
51	*Amaryllis belladonna*	Chile	B	1.65	42.00	25.43	128.2	77.7	37.29	1.1	3.4
81	*Crinum amabile*	Venezuela	B	5.42	211.5	38.99	266.9	49.2	25.86	8.2	10.4
93	*Crinum amabile*	Ecuador	B	2.21	60.69	27.46	111.3	50.3	20.57	2.9	5.4
**56**	***Crinum erubescens***	**Bolivia**	**B**	**9.50**	**234.7**	**24.69**	**678.3**	**71.4**	**11.10**	**21.1**	**61.1**
101	*Eucharis formosa*	Ecuador	B	9.71	346.7	35.71	778.9	80.2	26.93	12.9	28.9
23	*Rhodophiala andicola*	Chile	B	6.20	134.9	21.75	77.37	12.5	–	–	–
**24**	***Rhodophiala andicola***	**Chile**	**AP**	**6.13**	**228.4**	**37.27**	**188.1**	**30.7**	**10.18**	**22.4**	**18.5**

Extracts that showed specific anti-amastigote activity with SI > 10 with respect to Vero and HepG2 cell toxicity assays are highlighted in bold

*B* bulbs, *AP* aerial parts

^a^TC_50_ on Vero cells toxicity assay

^b^TC_50_ on HepG2 cells toxicity assay

^c^Anti-amastigote assay

### HepG2 cell toxicity assay

In order to anticipate potential liver toxicity in humans, we assessed the toxicity of those seven selected *T. cruzi*-specific extracts against the human cell line HepG2 [33, 34]. In this case, we included the reference drugs BNZ and DTX in every plate, which respectively showed average TC_50_ values of 197.8 (28.4) µM and 0.19 (0.06) µM [i.e. 51.47 (7.39) ppm and 0.14 (0.046) ppm] (Additional file 3: Figure S1B, C). Contrary to what was observed on Vero cell toxicity assay, toxicity of the extracts to HepG2 cells was lower than what was retrieved for BNZ (Table 1). Extract 23, however, showed a SI value < 20, and it was discarded from further progression. In contrast, extracts 51, 56 and 101 showed SI values > 70 due to their potent anti-*T. cruzi* activity and low toxicity (Table 1).

### Anti-amastigote activity of specifically active extracts

**Fig. 4:**
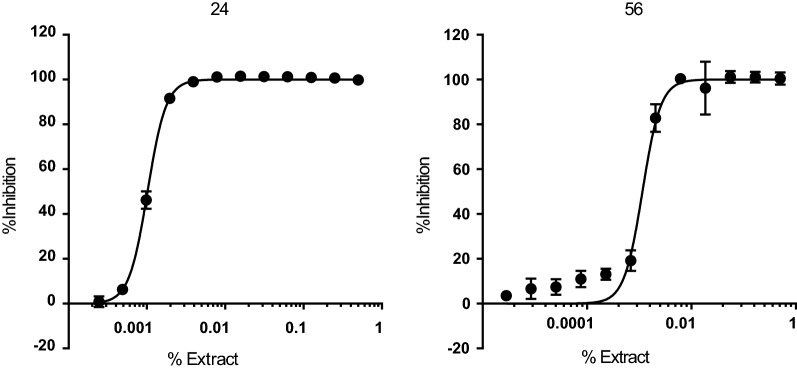
Dose–response curves of the extracts that had specific (SI > 10) anti-amastigote activity. Graphs represent mean results and SD of at least three replicates [23, 24, etc.]

Amastigotes are likely the main target for any prospective drug to treat chronic *T. cruzi* infections. Thus, we evaluated the activity of the six extracts that were specific (SI > 20) against *T. cruzi* and non-toxic to HepG2 cells by means of a biological assay specifically targeting the mammalian replicative form of the parasite. We included the reference drug BNZ in each assay round, registering an average IC_50_ value of 2.02 (0.09) µM [or 0.53 (0.02) ppm]. Generally, we found that all the extracts were less potent against the amastigote form than BNZ (Table 1). According to the threshold of IC_50_ < 30× that of BNZ, we selected two extracts (Table 1). Both had anti-amastigote SI values > 20 with respect to Vero cells and were not toxic to HepG2 cells (Fig. 4).

## Discussion

Anti-protozoal properties of Amaryllidaceae extracts, or alkaloids purified from them, have been evaluated before [21–26], but this is to the best of our knowledge the largest screening of a collection of extracts derived from plants of this family against a parasite pathogen. Upon screening of 79 extracts against *T. cruzi*, we found 37 active and 42 inactive extracts in the primary anti-parasitic assay. All extracts from genus *Ismene* were inactive, while all from genera *Eucharis*, *Hymenocallis* and *Pyrolirion* were initially active. Distinct anti-*T. cruzi* activities were retrieved with extracts from other genera, depending on the part of the plant processed. Variations in the alkaloid composition of different parts of the plant within the same species is explained by ontogenetic (seasonal) variability, as Amaryllidaceae alkaloids can vary among tissues during the plants' biological cycle [15]. In general, extracts obtained upon processing bulbs displayed higher activity against the parasite than those obtained from aerial parts or flowers. In fact, 29 out of the 37 active extracts were obtained from bulbs.

Active extracts showed a range of IC_50_ values between 1.65 and 11.23 ppm (≤ 30 × that of BNZ) and all were progressed onto the secondary Vero cell toxicity assay to determine their SI. Only seven extracts had a SI > 20, and a subsequent HepG2 cell assay determined that six had toxicity to HepG2 cells that was at least 20-fold lower than that against the parasite (Table 1). The observation that extracts were generally less toxic to HepG2 cells than to Vero cells is likely related to the exposure time of 2 or 4 days in the corresponding assays. Ultimately, just two out of those six displayed specific anti-amastigote activity (SI > 20): one from *Rhodophiala andicola* (number 24) and another from *Crinum erubescens* (number 56). Among the extracts that did not display specific anti-amastigote activity, some had been previously studied against *T. cruzi* and their alkaloid composition deciphered. For example, extract 51 from *Amaryllis belladonna* bulbs collected in Chile had the most potent IC_50_ value of all (IC_50_ = 1.65 ppm) and yielded SI rates of 25.43 and 77.69 against Vero and HepG2 cells (Table 1). A former study identified 26 different alkaloids in bulb samples from that species collected in Brazil and isolated three of them (1-*O*-acetylcaranine, 3-*O*-acetylhamayne and buphanamine) [22]. In vitro anti-protozoal assays revealed that the higher anti-*T. cruzi* activity was provided by 3-*O*-acetylhamayne despite its toxicity to L6 cells [22], and the same study reported that the crude extract was less toxic (TC_50_ = 34.3 µg/mL) than the alkaloid itself [22]. Expecting higher toxicity of isolated alkaloid species to parasites and host cells, we determined a SI threshold > 20 in the primary anti-parasitic screening, which is double what has been used in other studies [23, 30].

Regarding the two selected extracts, number 56 exhibited the sixth most potent anti-parasitic activity in the primary assay (IC_50_ = 9.50 ppm), and the second most potent against the amastigote form (IC_50_ = 11.10 ppm), highlighting its wide SI windows versus HepG2 cells (SI = 71.4 and 61.1, for the primary and anti-amastigote assays respectively). Notably, lower toxicity against Vero cells was observed with extract 56 obtained from bulbs in comparison to extract 82 from the same plant and site but processed from aerial parts. In relation to that, Rojas-Vera and co-workers found that the quantity of lycorine-type alkaloids was higher in bulbs from *C. erubescens* (collected in Venezuela) while non-identified alkaloids were higher in leaves [35]. It has been described that lycorine extracted from *Narcissus* species had an IC_50_ = 0.7 µM against *T. cruzi* but was not parasite-specific [23], which may suggest the presence of other alkaloids in extract 56 that are responsible for its registered anti-parasitic capacity. Notably, *C. erubescens* bulbs collected in Venezuela (extract number 83) had higher toxicity to Vero cells than extract 56, which could be due to variation in alkaloids among the same plants collected from different locations [18].

The alkaloid profile and anti-acetylcholinesterase activity of extracts from *Rhodophiala* species collected in Chile have been reported in previous studies [18]. Here we present for the first time results of its anti-*T. cruzi* properties. Some *Rhodophiala* species have been described as a rich source of lycorine, and haemanthamine/crinine- and tazettine-type alkaloids [18], which are some of the alkaloids that have shown activity against *T. cruzi* before [21, 23]. Herein, an aerial parts extract from *R. andicola* collected in Chile (extract number 24) had an IC_50_ value of 6.13 ppm in the primary assay and the most potent activity against amastigotes of all extracts tested (IC_50_ = 10.18 ppm), achieving good SI values. The alkaloid profile of this extract has been described by Tallini and co-workers, showing high quantities of tazettine and other non-identified type alkaloids [18]. The extract obtained upon processing the bulbs of this species (number 23) showed higher toxicity against both mammalian cell lines, resulting in SI values < 20, which might be explained by higher content of lycorine and haemanthamine/crinine type alkaloids [18].

Whether already known alkaloids or newly identified ones are responsible for the anti-*T. cruzi* activity observed in the two selected extracts will need to be further investigated. Upon identifying these alkaloids, specifically evaluating them against the *T. cruzi* Tulahuen β-galactosidase strain, as well as other parasite strains, with orthogonal anti-parasitic assays (e.g. imaging assays) will be of relevance for their progress in the drug discovery pathway [30]. Although there is little information on the structure-activity relationships (SAR) of Amaryllidaceae-derived alkaloids, some results suggest that the methylenedioxy group and the presence of a tertiary nitrogen without a methyl group can contribute to increasing their anti-parasitic activity [36]. Identification and functional characterization of any new alkaloids would widen current SAR knowledge, helping guide chemical modification to obtain higher potency and specific anti-parasitic outputs. Despite the difficulties and high attrition rates expected, the anti-*T. cruzi* activity rates reported in this work allow us to be optimistic and encourage further studies.

## Supplementary Information

**Additional file 1: Table S1.** Active extracts IC50, TC50 and SI parameters are shown for each extract. IC50 and TC50 R2 statistical indicator is also included in the table. Those extracts that presented specific activity (SI > 20) are highlighted in grey.

**Additional file 2: Table S2.** Inactive extracts IC50 values and their R2 curve adjustment indicator are included.

**Additional file 3: Figure S1.** Dose response curves of standard drug BNZ and DTX. (A) Anti-*T. cruzi* assay (circles), anti-amastigote assay (triangles) and Vero cell toxicity assay (squares) of BNZ. (B) HepG2 cell toxicity assays of BNZ and (C) DTX, respectively.

## Data Availability

Data and materials can be made available upon reasonable request to the authors.
