# Supplementary material for: Amaryllidaceae plants: a potential natural resource for the treatment of Chagas disease
Source: Parasit Vectors. 2021 Jun 26;14:337. doi: 10.1186/s13071-021-04837-9 (PMC8235838; doi:10.1186/s13071-021-04837-9)
Supplement: Supplementary file 1 — Additional file 1: Table S1. Active extracts IC50, TC50 and SI parameters are shown for each extract. IC50 and TC50 R2 statistical indicator is also included in the table. Those extracts that presented specific activity (SI > 20) are highlighted in grey. [file 13071_2021_4837_MOESM1_ESM.docx]

Table S1. Active extracts IC_50_, TC_50_ and SI parameters are shown for each extract. IC_50_ and TC_50_ R^2^ statistical indicators are also included in the table. Those extracts that presented specific activity (SI > 20) are highlighted in grey. (Table S1 continues in next page).

| No. extract | Plant species of origin | Plant part^*^ | Country of collection | IC_50_ (ppm) | R^2^ | TC_50_ (ppm) | R^2^ | SI |
| --- | --- | --- | --- | --- | --- | --- | --- | --- |
| 87 | *Amaryllis belladona* | B | Venezuela | 5.49 | 0.989 | 46.12 | 0.946 | 8.40 |
| 51 | *Amaryllis belladona* | B | Chile | 1.65 | 0.973 | 41.97 | 0.943 | 25.43 |
| 53 | *Amaryllis belladonna* | SR | Chile | 6.40 | 0.997 | 124.80 | 0.952 | 19.49 |
| 109 | *Clinanthus sp.* | B | Peru | 8.75 | 0.996 | 88.28 | 0.946 | 10.09 |
| 110 | *Clinanthus sp.* | B | Peru | 9.64 | 0.972 | 108.80 | 0.823 | 11.28 |
| 111 | *Clinanthus sp.* | L | Peru | 8.86 | 0.999 | 88.00 | 0.890 | 9.94 |
| 80 | *Crinum amabile* | L | Venezuela | 7.32 | 0.984 | 50.10 | 0.927 | 6.85 |
| 81 | *Crinum amabile* | B | Venezuela | 5.42 | 0.991 | 211.50 | 0.948 | 38.99 |
| 93 | *Crinum amabile* | B | Ecuador | 2.21 | 0.992 | 60.69 | 0.867 | 27.46 |
| 83 | *Crinum erubescens* | B | Venezuela | 4.93 | 0.997 | 56.99 | 0.946 | 11.57 |
| 56 | *Crinum erubescens* | B | Bolivia | 9.50 | 0.996 | 234.70 | 0.920 | 24.69 |
| 58 | *Crinum erubescens* | AP | Bolivia | 7.49 | 0.974 | 84.73 | 0.918 | 11.31 |
| 85 | *Crinum moorie* | B | Venezuela | 7.48 | 0.970 | 63.15 | 0.918 | 8.44 |
| 99 | *Crinum powelli* | WP | hybrid | 11.23 | 0.995 | 59.48 | 0.913 | 5.30 |
| 100 | *Eucharis astrophiala* | B | Ecuador | 5.22 | 0.993 | 30.46 | 0.938 | 5.83 |
| 101 | *Eucharis formosa* | B | Ecuador | 9.71 | 0.988 | 346.70 | 0.799 | 35.71 |
| 104 | *Eucharis ruthiana* | B | Ecuador | 7.52 | 0.988 | 57.40 | 0.942 | 7.64 |
| 107 | *Hymenocallis tubiflora* | B | Ecuador | 5.86 | 0.990 | 45.92 | 0.808 | 7.84 |
| 117 | *Narcissus cv Icefolis* | WP | cultivar | 2.77 | 0.991 | 17.25 | 0.881 | 6.22 |
| 116 | *Narcissus papyraceus* | WP | Spain | 4.97 | 0.991 | 53.94 | 0.905 | 10.86 |
| 96 | *Phaedranassa cinerea* | B | Ecuador | 4.65 | 0.989 | 40.79 | 0.861 | 8.76 |
| 102 | *Phaedranassa cuencana* | B | Ecuador | 4.14 | 0.991 | 42.36 | 0.950 | 10.23 |
| 103 | *Phaedranassa cuencana* | B | Ecuador | 7.14 | 0.995 | 43.12 | 0.917 | 6.04 |
| 95 | *Phaedranassa dubia* | B | Ecuador | 5.75 | 0.985 | 92.35 | 0.818 | 16.06 |
| 91 | *Phaedranassa glauciflora* | B | Ecuador | 4.49 | 0.998 | 33.17 | 0.841 | 7.39 |
| 105 | *Phaedranassa cuencana* | B | Ecuador | 4.74 | 0.964 | 40.82 | 0.834 | 8.62 |
| No. extract | **Plant species of origin** | **Plant part^*^** | **Country of collection** | **IC_50_ (ppm)** | **R^2^** | **TC_50_ (ppm)** | **R^2^** | **SI** |
| 106 | *Phaedranassa cuencana* | B | Ecuador | 7.02 | 0.995 | 21.41 | 0.936 | 3.05 |
| 90 | *Phaedranassa tunguraguae* | B | Ecuador | 6.60 | 0.998 | 51.42 | 0.901 | 7.80 |
| 45 | *Phycella australis* | B | Chile | 9.27 | 0.988 | 46.58 | 0.893 | 5.03 |
| 112 | *Pyrolirion flammeum* | B | Peru | 5.49 | 0.995 | 56.61 | 0.928 | 10.32 |
| 23 | *Rhodophiala andicola* | B | Chile | 6.20 | 0.995 | 134.90 | 0.957 | 21.75 |
| 24 | *Rhodophiala andicola* | AP | Chile | 6.13 | 0.997 | 228.40 | 0.963 | 37.27 |
| 36 | *Rhodophiala andicola* | B | Chile | 10.64 | 0.970 | 47.92 | 0.915 | 4.50 |
| 25 | *Rhodophiala splendens* | B | Chile | 11.20 | 0.979 | 58.44 | 0.919 | 5.22 |
| 97 | *Stenomesson campanulatum* | B | Peru | 1.67 | 0.986 | 23.61 | 0.920 | 14.11 |
| 60 | *Zephyranthes andina* | B | Bolivia | 8.74 | 0.995 | 171.70 | 0.926 | 19.65 |
| 89 | *Zephyranthes carinata* | B | Venezuela | 4.83 | 0.979 | 51.83 | 0.947 | 10.72 |

^*^AP: aerial parts; B: bulbs; F: flowers; L: leaves; SR: small roots; WP: whole plant.
