# Supplementary material for: Amaryllidaceae plants: a potential natural resource for the treatment of Chagas disease
Source: Parasit Vectors. 2021 Jun 26;14:337. doi: 10.1186/s13071-021-04837-9 (PMC8235838; doi:10.1186/s13071-021-04837-9)
Supplement: Supplementary file 2 — Additional file 2: Table S2. Inactive extracts IC50 values and their R2 curve adjustment indicator are included. [file 13071_2021_4837_MOESM2_ESM.docx]

Table S2. Inactive extracts IC_50_ values; R^2^ curve adjustment indicator is also included for each extract.

| No. extract | Plant species of origin | Part of the plant^*^ | Country of collection | IC_50_ (ppm) | R^2^ |
| --- | --- | --- | --- | --- | --- |
| 86 | *Amaryllis belladona* | L | Venezuela | 16.95 | 0.993 |
| 52 | *Amaryllis belladona* | AP | Chile | 13.13 | 0.993 |
| 4 | *Clinanthus incarnatum* | B | Ecuador | 12.17 | 0.997 |
| 2 | *Clinanthus milagroanthus* | B | Peru | 12.06 | 0.979 |
| 8 | *Crinum amabile* | B | Ecuador | 22.15 | 0.993 |
| 94 | *Crinum amabile* | L | Ecuador | 18.81 | 0.978 |
| 59 | *Crinum augustum* | B | Bolivia | 204.90 | 0.979 |
| 82 | *Crinum erubescens* | L | Venezuela | 19.37 | 0.990 |
| 57 | *Crinum erubescens* | AP | Bolivia | 14.92 | 0.996 |
| 84 | *Crinum moorie* | L | Venezuela | 181.40 | 0.934 |
| 92 | *Eucrosia mirabillis* | B | Ecuador | 212.40 | 0.994 |
| 1 | *Ismene amancaes* | B | Peru | 706.9 | 0.932 |
| 3 | *Ismene amancaes* | F | Peru | 40.81 | 0.992 |
| 113 | *Ismene amancaes* | L | Peru | 22.85 | 0.987 |
| 115 | *Narcissus cantabricus* | WP | Spain | 32.87 | 0.977 |
| 55 | *Narcissus assoanus* | WP | Spain | 6,255 | 0.961 |
| 108 | *Phaedranassa brevifolia* | B | Ecuador | 15.34 | 0.996 |
| 5 | *Phaedranassa cinerea* | B | Ecuador | 50.25 | 0.979 |
| 6 | *Phaedranassa dubia* | B | Ecuador | 36.73 | 0.989 |
| 46 | *Phycella australis* | AP | Chile | 17.69 | 0.992 |
| 47 | *Phycella australis* | SR | Chile | 58.93 | 0.985 |
| 37 | *Rhodophiala andicola* | AP | Chile | 23.13 | 0.994 |
| 42 | *Rhodophiala andicola* | B | Chile | 16.17 | 0.997 |
| 43 | *Rhodophiala andicola* | AP | Chile | 20.56 | 0.990 |
| 44 | *Rhodophiala andicola* | SR | Chile | 16.52 | 0.971 |
| 40 | *Rhodophiala araucana* | B | Chile | 24.46 | 0.991 |
| 41 | *Rhodophiala araucana* | AP | Chile | 353.00 | 0.990 |
| 38 | *Rhodophiala montana* | B | Chile | 155.50 | 0.998 |
| 39 | *Rhodophiala montana* | AP | Chile | 146.20 | 0.991 |
| No. extract | **Plant species of origin** | **Part of the plant^*^** | **Country of collection** | **IC_50_ (ppm)** | **R^2^** |
| 27 | *Rhodophiala pratensis* | B | Chile | 72.70 | 0.996 |
| 28 | *Rhodophiala pratensis* | AP | Chile | 36.86 | 0.994 |
| 29 | *Rhodophiala pratensis* | B | Chile | 85.83 | 0.982 |
| 30 | *Rhodophiala pratensis* | AP | Chile | 1,132 | 0.988 |
| 31 | *Rhodophiala pratensis* | B | Chile | 62.29 | 0.991 |
| 32 | *Rhodophiala pratensis* | AP | Chile | 12.67 | 0.993 |
| 33 | *Rhodophiala pratensis* | SR | Chile | NA | 0.862 |
| 34 | *Rhodophiala pratensis* | B | Chile | 197.20 | 0.998 |
| 35 | *Rhodophiala pratensis* | AP | Chile | 303.90 | 0.991 |
| 26 | *Rhodophiala splendens* | AP | Chile | 28.90 | 0.978 |
| 7 | *Stenomesson aurantiacum* | B | Ecuador | 303.60 | 0.989 |
| 61 | *Zephyranthes andina* | AP | Bolivia | 120.20 | 0.990 |
| 88 | *Zephyranthes carinata* | L | Venezuela | 24.23 | 0.996 |

^*^AP: aerial parts; B: bulbs; SR: small roots; WP: whole plant. NA, not adjusted.
