# Supplementary figures and images for: Amaryllidaceae plants: a potential natural resource for the treatment of Chagas disease
Source: Parasit Vectors. 2021 Jun 26;14:337. doi: 10.1186/s13071-021-04837-9 (PMC8235838; doi:10.1186/s13071-021-04837-9)

**A**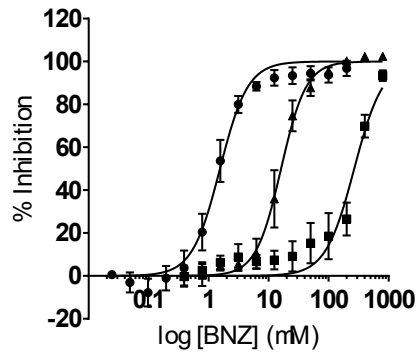**B**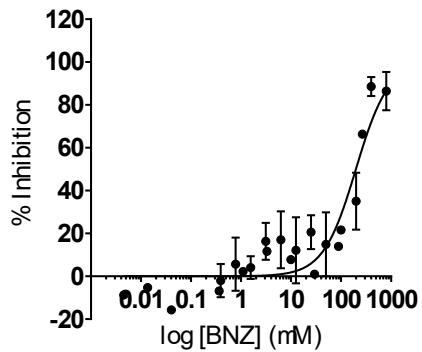**C**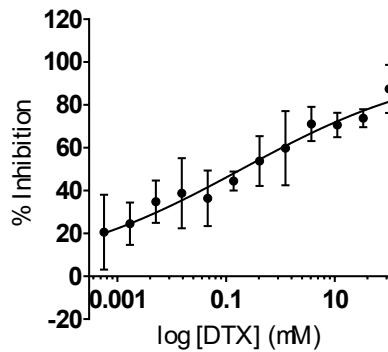

Supplement: Supplementary file 3 — Additional file 3: Figure S1. Dose response curves of standard drug BNZ and DTX. (A) Anti-T. cruzi assay (circles), anti-amastigote assay (triangles) and Vero cell toxicity assay (squares) of BNZ. (B) HepG2 cell toxicity assays of BNZ and (C) DTX, respectively. [file 13071_2021_4837_MOESM3_ESM.pdf]
